# The complete mitochondrial genome data of the Common Rose butterfly, *Pachliopta aristolochiae* (Lepidoptera, Papilionoidea, Papilionidae) from Malaysia

**DOI:** 10.1016/j.dib.2021.107740

**Published:** 2021-12-23

**Authors:** Marylin Miga, Puteri Nur Syahzanani Jahari, Chan Vei Siang, Kamarul Rahim Kamarudin, Mohd Shahir Shamsir, Lili Tokiman, Sivachandran Parimannan, Heera Rajandas, Farhan Mohamed, Faezah Mohd Salleh

**Affiliations:** a Department of Biosciences, Faculty of Science, Universiti Teknologi Malaysia, Johor Bahru, Johor 81310, Malaysia; b School of Computing, Faculty of Engineering, Universiti Teknologi Malaysia, Johor Bahru, Johor 81310, Malaysia; c Centre of Research for Sustainable Uses of Natural Resources (SUNR), Faculty of Applied Sciences and Technology, Universiti Tun Hussein Onn Malaysia, Pagoh Higher Education Hub, Muar, Johor 84600, Malaysia; d Johor National Parks Corporation, Kota Iskandar, Iskandar Puteri, Johor 79575, Malaysia; e Centre of Excellence for Omics-Driven Computational Biodiscovery (COMBio), Faculty of Applied Sciences, AIMST University, Bedong, Kedah 08100, Malaysia; f Deakin Genomics Centre, School of Life and Environmental Sciences, Faculty of Science, Engineering and Built Environment, Deakin University, Waurn Ponds Campus, Victoria 3216, Australia

**Keywords:** *Pachliopta aristolochiae*, Mitogenome, Lepidoptera, Papilionidae, Malaysia

## Abstract

Here, we present the complete mitochondrial genome of *Pachliopta aristolochiae*, a Common Rose butterfly from Malaysia. The sequence was generated using Illumina NovaSeq 6000 sequencing platform. The mitogenome is 15,235bp long, consisting of 13 protein-coding genes, 22 transfer RNAs, two ribosomal RNAs, and two D-loop regions. The total base composition was (81.6%), with A (39.3%), T (42.3%), C (11.0%) and G (7.3%). The gene order of the three tRNAs was *trnM-trnI-trnQ*, which differs from the ancestral insect gene order *trnI-trnQ-trnM*. Phylogenetic tree analysis revealed that the sequenced *Pachliopta aristolochiae* in this data is closely related to *Losaria neptunus* (NC 037868), with highly supported ML and BI analysis. The data presented in this work can provide useful resources for other researchers to study deeper into the phylogenetic relationships of Lepidoptera and the diversification of the *Pachliopta* species. Also, as one of the bioindicator species, this data can be used to assess environmental changes in the terrestrial and aquatic ecosystem via enviromental DNA approahes. The mitogenome of *Pachliopta aristolochiae* is available in GenBank under the accession number MZ781228.

## Specifications Table

**Table:** 

Subject	Genomics
Specific subject area	Lepidoptera, Papilionidae, Mitogenomics
Type of data	• Fasta: Mitogenome sequence data • Tables: Sequencing data, gene features, base composition, list of Lepidoptera mitogenomes used for phylogenetic analyses • Figures: Circular mitogenome map, features of the D-loop regions, phylogenetic tree analysis
How the data were acquired	Whole genome shotgun sequencing using Illumina NovaSeq 6000 platform with 150 paired-end mode (PE150)
Data format	Raw and analyzed
Parameters for data collection	Genomic DNA was extracted from fresh tissue sample of *Pachliopta aristolochiae* using the Qiagen Blood and Tissue Kit (Qiagen, Valencia, CA) and fragmented using a Bioruptor® system. The library was prepared using NEBNext® Ultra™ II DNA Library Prep Kit for Illumina®. The sample was then sent for sequencing using the Illumina NovaSeq 6000 platform with 150 paired-end mode (PE150).
Description of data collection	The assembly was done using NOVOPlasty v.4.2 and run through a PALEOMIX BAM pipeline to assess the mitogenome mapping. Annotation was done using the MITOS v2 web server and the predicted protein-coding genes were further verified using the Open Reading Frame (ORF) Finder. The circular mitogenome map was generated using OGDRAW. PhyloSuite v1.2.2 was used to extract, align and concatenate 13 protein-coding genes from 22 Lepidoptera mitogenomes prior to phylogenetic analysis. IQ-Tree and MrBayes v3.2.7 programs were used to build the phylogenetic trees using Maximum-Likelihood (ML) and Bayesian Inference (BI) probability method. PartitionFinder v2.2.1 was used to set the best partitioning schemes for the dataset. The resulting phylogenetic trees were visualized using Figtree v1.4.4.
Data source location	The sample *Pachliopta aristolochiae* (voucher no: DIB022) was collected from Sungai Semawak Taman Negara Endau-Rompin Johor, Malaysia (5.62 N, 100.46 E) in March 2019.
Data accessibility	Repository name: NCBI BioProject Data identification number: PRJNA753627 Direct URL to data: http://www.ncbi.nlm.nih.gov/bioproject/753627 Repository name: NCBI GenBank Data identification number: MZ781228 Direct URL to data: https://www.ncbi.nlm.nih.gov/nuccore/mz781228 Repository name: Mendeley Data Data identification number: 10.17632/n52pmth7cc.2 Direct URL to data: https://data.mendeley.com/datasets/n52pmth7cc/2

## Value of the Data

• The sequenced mitochondrial genome of the Common Rose butterfly, *Pachliopta aristolochiae* in this data represents the *Pachliopta* species originating from Malaysia.
• As one of the bioindicator species, this mitogenome data can be used to assess environmental changes in the terrestrial and aquatic ecosystem via environmental DNA approaches.
• The additional mitogenome data of *Pachliopta aristolochiae* generated can also provide the relevant information needed for other researchers to study deeper into the phylogenetic relationships of Lepidoptera and the diversification of the *Pachliopta* species.

## 1 Data Description

**Fig. 1:**
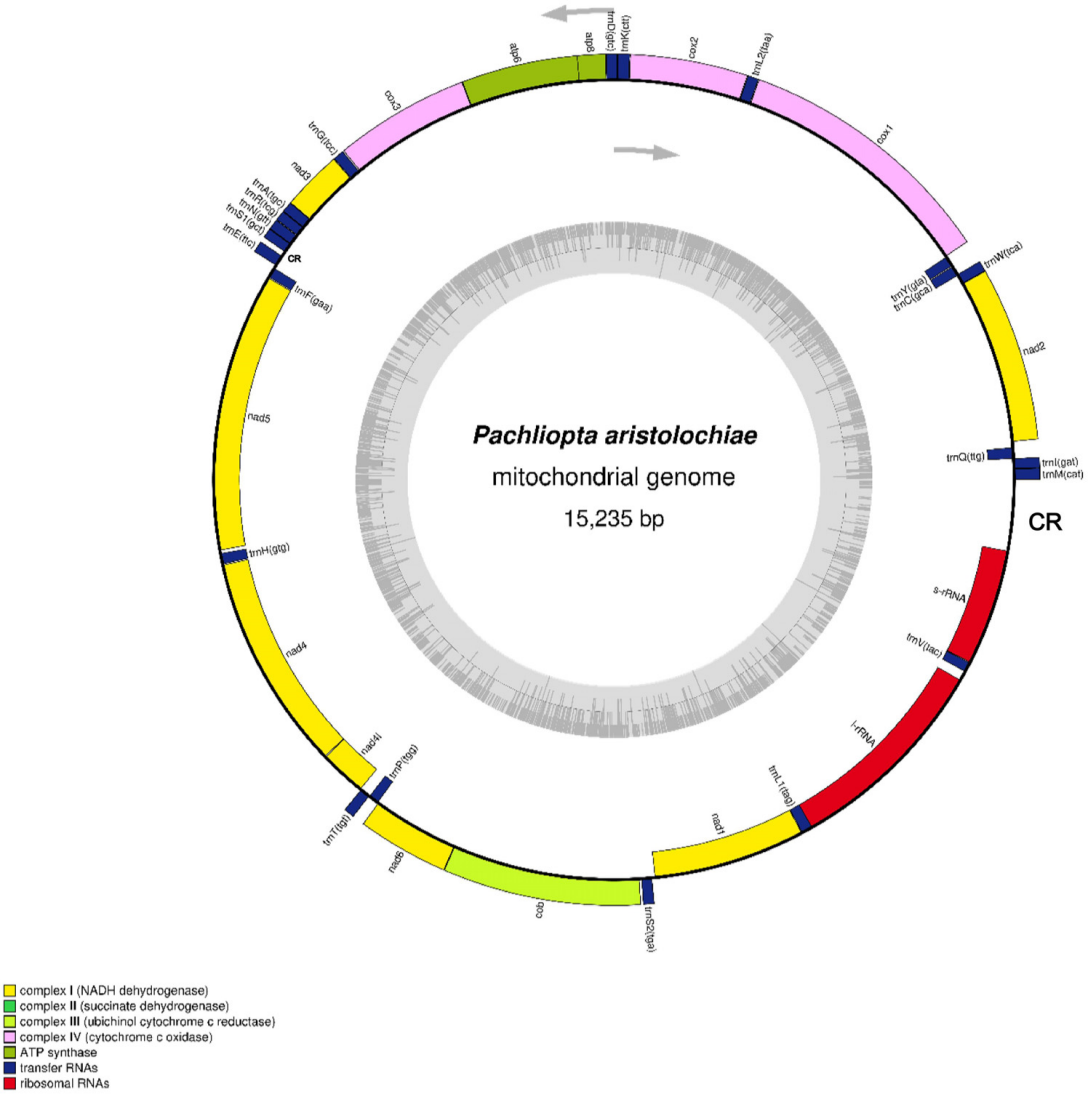
Mitogenome map of *Pachliopta aristolochiae* generated using OGDRAW [3]. The genes scattered on the heavy strand are shown on the outer side of the circle, while the inner side shows those that are scattered on the light strand. The arrows indicate the direction of gene transcription. CR represents the control region (D-loop).

The Common Rose butterfly, *Pachliopta aristolochiae* mitogenome is a circular DNA with a total of 15,235bp in length (Fig. 1). Table 1 shows the statistical data information for the sequence reads. The mitogenome encodes 13 protein-coding genes (PCGs), 22 transfer RNAs, 2 ribosomal RNAs, and two D-loop regions (Table 2). The gene order of *P.aristolochiae* located between the D-loop and NAD2 was *trnM-trnI-trnQ*, which had been observed in most Lepidoptera mitogenomes, however, it differs from that of the ancestral insect gene order, *trnI-trnQ-trnM* [1]. The total size of the PCGs was 11,178bp in length and the tRNAs were 1,452bp long, ranging from 60bp to 71bp. Meanwhile, the sizes for the 12S and 16S RNAs are 719bp and 1280bp respectively. The majority of the PCGs (NAD2, COX1, COX2, ATP8, ATP6, COX3, NAD3, NAD6, CYTB) are scattered on the heavy strand, and NAD5, NAD4, NAD4l, NAD1 are on the light strand. Out of 13 PCGs, 12 were initiated by the typical ATN codon except for COX1 which uses the CGA start codon. Contrary to the start codon, two PCGs (COX2 and NAD4) were terminated with the incomplete stop codon T and the others were terminated by either TAA or TAG stop codon. The phenomena of incomplete termination codon had been observed in most Lepidoptera mitogenomes, and are associated with the polyadenylation process [2]. The mitogenome of *P. aristolochiae* showed an AT content of 81.64% with the base composition of A (39.3%), T (42.3%), C (11.0%), and G (7.3%) as shown in Table 3. The nucleotide skew statistics of the whole mitogenome indicates a high occurrence of T over A, and C over G with an AT-skew of -0.037 and GC-skew of -0.202.

**Table 1:** Sequencing data of *Pachliopta aristolochiae* mitogenome.

	Pachliopta aristolochiae
Raw reads	10,102,746
Trimmed reads	10,102,675
Ave. read length	149.5
Mapped reads	17,890
% mapped reads	0.002
Depth of coverage (X)	175.72

**Table 2:** Gene features of *Pachliopta aristolochiae* mitogenome.

	Position				
Gene (anticodon)	Start	Stop	Direction	Size	Start/Stop codon
trnM(cat)	1	67	F	67	
trnI(gat)	67	130	F	64	
trnQ(ttg)	128	196	R	69	
NAD2	231	1244	F	1014	ATT/TAA
trnW(tca)	1243	1307	F	65	
trnC(gca)	1300	1365	R	66	
trnY(gta)	1368	1434	R	67	
COX1	1437	2967	F	1531	CGA/TAA
trnL2(taa)	2968	3034	F	67	
COX2	3035	3716	F	682	ATG/T
trnK(ctt)	3717	3787	F	71	
trnD(gtc)	3787	3853	F	67	
ATP8	3854	4021	F	168	ATT/TAA
ATP6	4015	4692	F	678	ATG/TAA
COX3	4692	5477	F	786	ATG/TAA
trnG(tcc)	5481	5546	F	66	
NAD3	5547	5900	F	354	ATA/TAG
trnA(tgc)	5899	5963	F	65	
trnR(tcg)	5963	6024	F	62	
trnN(gtt)	6025	6089	F	65	
trnS1(gct)	6089	6148	F	60	
D-loop	6148	6192	F	45	
trnE(ttc)	6178	6246	F	69	
trnF(gaa)	6265	6330	R	66	
NAD5	6333	8048	R	1716	ATT/TAA
trnH(gtg)	8067	8133	R	67	
NAD4	8137	9472	R	1336	ATG/T
NAD4l	9474	9764	R	291	ATG/TAA
trnT(tgt)	9767	9831	F	65	
trnP(tgg)	9832	9896	R	65	
NAD6	9899	10432	F	534	ATT/TAA
CYTB	10432	11580	F	1149	ATG/TAA
trnS2(tga)	11593	11657	F	65	
NAD1	11674	12612	R	939	ATG/TAA
trnL1(tag)	12613	12683	R	71	
16S rRNA	12659	13963	R	1280	
trnV(tac)	14021	14083	R	63	
12S rRNA	14084	14802	R	719	
D - loop	14816	15235	F	420

**Table 3:** Base composition and AT/GC skewness for each gene region of *Pachliopta aristolochiae* mitogenome.

Gene	Size (bp)	A%	G%	T%	C%	A+T%	AT skew	GC skew
Whole mitogenome	15,235	39.3	7.3	42.3	11.0	81.6	−0.037	−0.202
Protein coding	11,178	33.5	10.1	46.8	9.6	80.3	−0.166	0.025
tRNA	1,452	43.0	10.5	39.1	7.5	82.1	0.048	0.167
rRNA	2,024	43.6	10.4	40.8	5.2	84.4	0.033	0.333
D-loop (major)	365	46.3	1.6	49.6	2.5	95.9	−0.034	−0.220
D-loop (minor)	45	46.7	2.2	51.1	0.0	97.8	−0.045	1.000

**Fig. 2:**
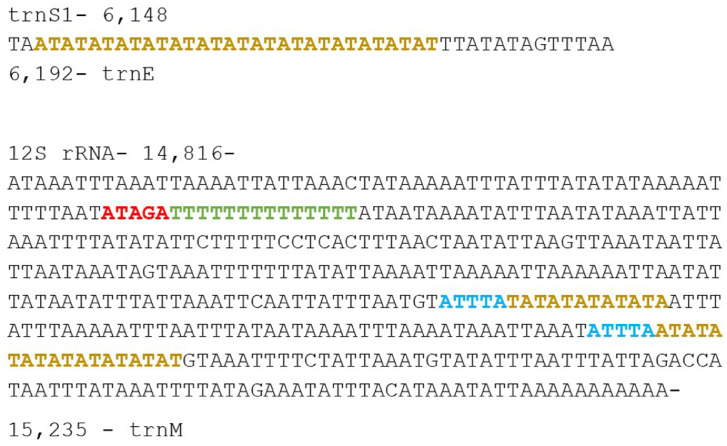
Features of the two D-loop regions of *Pachliopta aristolochiae* mitogenome located between trnS1 and trnE, as well as 12S rRNA and trnM. Conserved motifs ‘ATAGA’ and ‘ATTTA’ are indicated in red and blue respectively. Poly-T stretch is indicated in green while microsatellite-like elements (TA)n and (AT)n are shown in yellow.

Two D-loop regions were found in the sequenced mitogenome of *P.aristolochiae* for this data. The first region was found at the position 6148bp to 6192bp, located between trnS1 and trnE. This region is 45bp long, which contained a string of microsatellite-like element (AT). Meanwhile, the second D-loop region was 420bp long, located between 12S rRNA and trnM, spanning a conserved ATAGA motif, followed by a poly-T stretch, and a microsatellite-like element (AT)₉ and (TA)₆ after the motif ATTTA, as commonly found in all Lepidoptera mitogenomes [4]. Fig. 2 describe the features of the two D-loop regions.

**Fig. 3:**
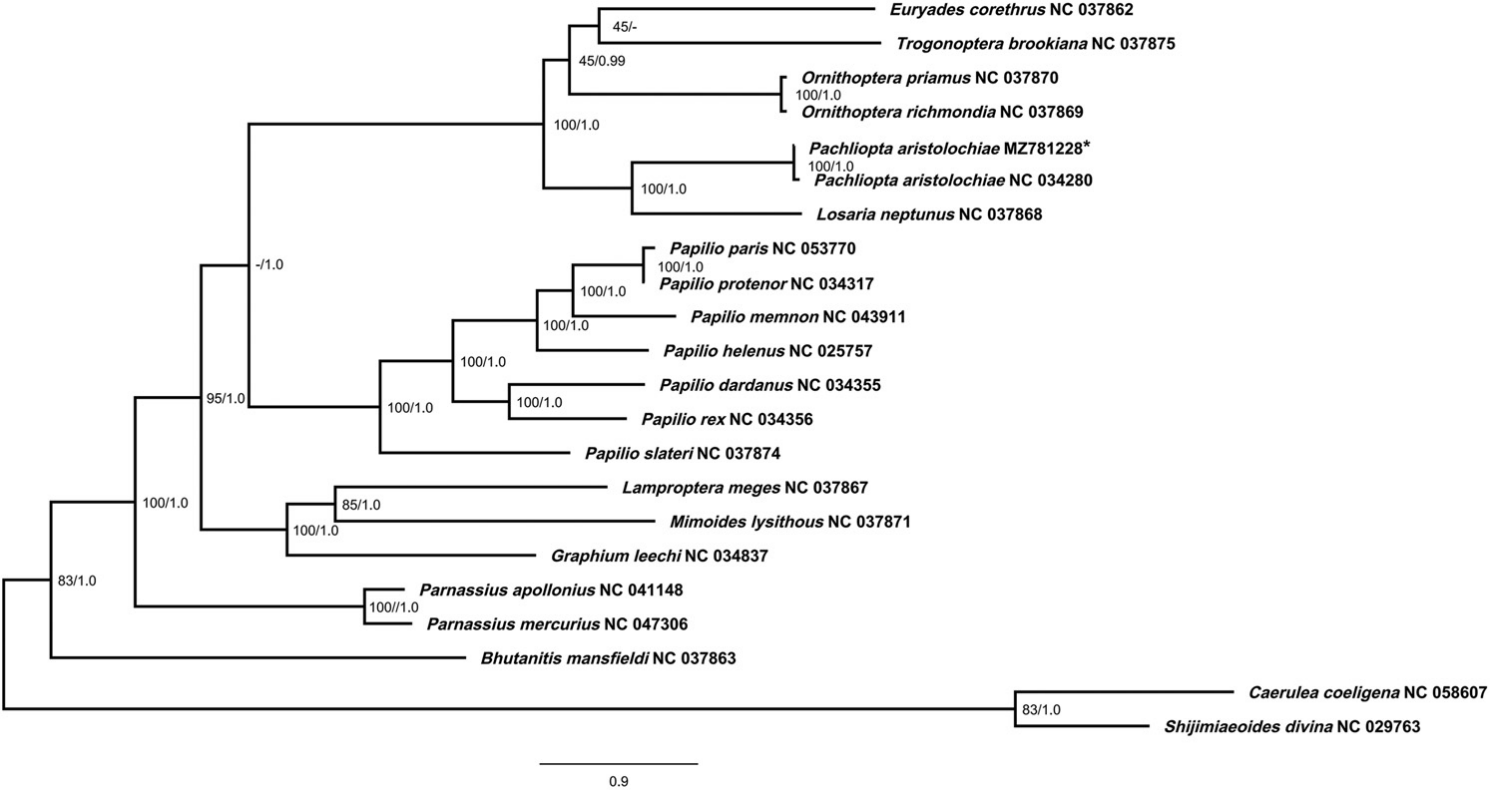
Phylogenetic tree of *Pachliopta aristolochiae* (MZ781228), indicated by asterisk (*) and 21 other Lepidoptera mitogenomes built using Maximum-Likelihood (ML) and Bayesian Inference (BI) approach. Bootstrap support values were indicated on each tree node, showing the results of ML and BI analysis. *Caerulea coeligena* (NC 058607) and *Shijimiaeoides divina* (NC 029763) from the family Lycaenidae were used as outgroups.

Maximum-Likelihood (ML) and Bayesian Inference (BI) probability tree were generated using 13 PCGs of 22 Lepidoptera mitogenomes from the family Papilionidae and Lycaenidae obtained from GenBank, including the sequenced *P. aristolochiae* in this data (Table 4). The resulting trees yielded identical topology under the ML and BI analysis (Fig. 3). Most of the nodes are highly supported with bootstrap value of more than 70% in ML analysis, and a Bayesian posterior probability of more than 0.95 in BI analysis. The sequence *P. aristolochiae* (MZ781228) in this study is clustered with the previously sequenced *P. aristolochiae* (NC 034280) and are closely related to *Losaria neptunus* (NC 037868), supported with a bootstrap value of 100% in ML and 1.0 posterior probability value in BI. A BLASTn analysis was also conducted to compare between the two mitogenomes of *P.aristolochiae*, where *P.aristolochiae* (MZ781228) in this data is 99.42% similar to *P. aristolochiae* (NC 034280) deposited in GenBank.

**Table 4:** Lepidoptera mitogenomes used to build the phylogenetic tree analysis. The sequenced *P.aristolochiae* in this data is indicated by (*), with GenBank Accession No. MZ781228.

Family	Subfamily	Species	GenBank Accession No.
Papilionidae	Papilioninae	*Papilio paris*	NC 053770
Papilionidae	Parnassiinae	*Parnassius mercurius*	NC 047306
Papilionidae	Papilioninae	*Papilio memnon*	NC 043911
Papilionidae	Parnassiinae	*Parnassius apollonius*	NC 041148
Papilionidae	Papilioninae	*Pachliopta aristolochiae*	NC 034280
Papilionidae	Papilioninae	*Papilio protenor*	NC 034317
Papilionidae	Papilioninae	*Papilio dardanus*	NC 034355
Papilionidae	Papilioninae	*Papilio rex*	NC 034356
Papilionidae	Papilioninae	*Graphium leechi*	NC 034837
Papilionidae	Papilioninae	*Papilio helenus*	NC 025757
Papilionidae	Papilioninae	*Euryades corethrus*	NC 037862
Papilionidae	Parnassiinae	*Bhutanitis mansfieldi*	NC 037863
Papilionidae	Papilioninae	*Lamproptera meges*	NC 037867
Papilionidae	Papilioninae	*Losaria neptunus*	NC 037868
Papilionidae	Papilioninae	*Ornithoptera richmondia*	NC 037869
Papilionidae	Papilioninae	*Ornithoptera priamus*	NC 037870
Papilionidae	Papilioninae	*Mimoides lysithous*	NC 037871
Papilionidae	Papilioninae	*Papilio slateri*	NC 037874
Papilionidae	Papilioninae	*Trogonoptera brookiana*	NC 037875
Papilionidae	Papilioninae	Pachliopta aristolochiae*	MZ781228
Lycaenidae	Polyommatinae	*Caerulea coeligena*	NC 058607
Lycaenidae	Polyommatinae	*Shijimiaeoides divina*	NC 029763

## 2 Experimental Design, Materials and Methods

### 2.1 Sample collection, DNA extraction and pre-processing

The sample *Pachliopta aristolochiae* (voucher no: DIB022) was collected from Sungai Semawak Taman Negara Endau-Rompin Johor, Malaysia (5.62 N, 100.46 E) in March 2019. The genomic DNA was extracted from a fresh tissue sample using Qiagen Blood and Tissue Kit (Qiagen, Valencia, CA) and was fragmented using a Bioruptor® system [5]. The library preparation was done using NEBNext® Ultra™ II DNA Library Prep Kit for Illumina®, following the manufacturer's instructions. Then, the library was sent for sequencing using the Illumina NovaSeq 6000 platform with 150 paired-end mode (PE150). A total of 10,102,764 raw reads were obtained and firstly verified using the FastQC program for quality assessment (https://www.bioinformatics.babraham.ac.uk/projects/fastqc/). Next, the raw reads were trimmed for sequencing adapters, low-quality bases as well as Ns [6,7] using AdapterRemoval v2.3.2 [8]. Sequences with quality score of 20 and above were retained. Both the forward and reverse reads were interleaved into a single file before using PALEOMIX [9].

### 2.2 Mitogenome assembly, annotation and sequence analysis

The mitogenome was assembled using the NOVOPlasty v.4.2 [10] program with the default parameter. The reference sequence and seed input were taken from BOLD public data (http://barcodinglife.org/), with the sequence ID BKKP127-18.COI-5P. Next, the assembled mitogenome was run through PALEOMIX BAM pipeline [9] using default parameters to remove reads shorter than 15 bp after trimming. The mitogenome annotation was carried out using MITOS v2 web server [11], with reference set ‘RefSeq 81 Metazoa’ and genetic code ‘5’ for invertebrates. Then, the predicted proteins were verified using the Open Reading Frame (ORF) Finder (https://www.ncbi.nlm.nih.gov/orffinder/) server using BLASTP. To improve the genome annotation, the predicted proteins from MITOS v2 web server [11] and ORF Finder were aligned with the reference sequence of *Pachliopta aristolochiae* (NC 034280) in GenBank using Jalview 2 v11.1.4 [12]. Tablet software [13] was used to manually check for insertion and deletion of bases, as well as the sequence coverage. The total base compositions were calculated using BioEdit [14]. The AT/GC skewness was calculated as follows: AT skew= (A-T)/(A+T) and GC skew=(G-C)/(G+C), where each letter represents the total percentage of the respective base count. The annotated mitogenome sequence file was converted into GenBank format using GB2sequin web application [15]. The GenBank file format was then used to generate the circular mitogenome map using OGDRAW [3].

### 2.3 Phylogenetic analysis

A total of 21 available Lepidoptera mitogenomes from the family Papilionidae and Lycaenidae were obtained from GenBank (Table 4). *Caerulea coeligena* (NC 058607) and *Shijimiaeoides divina* (NC 029763) from the family Lycaenidae were used as outgroups. The PCGs of each Lepidoptera mitogenomes were firstly extracted using the PhyloSuite v1.2.2 [16] platform. The 13 protein-coding genes were then aligned in batches using the MAFFT program integrated into PhyloSuite [16] and were concatenated. Phylogenetic analyses were performed using Maximum-Likelihood (ML) and Bayesian Inference (BI) approach using the IQ-Tree [17] program implemented in PhyloSuite v1.2.2 [16] and MrBayes v3.2.7 [18] respectively. PartitionFinder v2.1.1 [19] was used to determine the best partitioning schemes for the dataset. Maximum-Likelihood (ML) tree was built using 5000 ultrafast bootstrapping with 1000 iterations, and the best substitution model was determine by PartitionFinder v2.1.1 [19]. For Bayesian Inference (BI) analysis, each partition was set to the GTR substitution model (nst=6) with gamma distributed rate variation across sites (rates=invgamma) and a proportion of invariable sites (GTR + Γ + I). The analysis was carried out for 10,000,000 generations with 4 chains, sampled every 1000 generations with a burn-in of 25% until the average standard deviation of split frequencies are less than 0.01. Tracer v1.7.2 was used to ensure sufficient parameter sampling and that the Estimated Sample Size (ESS) is more than 200 [20]. Both resulting trees were visualized using Figtree v1.4.4 (http://tree.bio.ed.ac.uk/software/figtree/).

## CRediT authorship contribution statement

**Marylin Miga:** Conceptualization, Methodology, Data curation, Software, Validation, Writing – original draft. **Puteri Nur Syahzanani Jahari:** Data curation, Conceptualization, Methodology, Software, Validation, Writing – review & editing. **Chan Vei Siang:** Methodology, Software. **Kamarul Rahim Kamarudin:** Methodology. **Mohd Shahir Shamsir:** Methodology, Formal analysis, Resources, Funding acquisition. **Lili Tokiman:** Methodology. **Sivachandran Parimannan:** Formal analysis, Resources, Funding acquisition. **Heera Rajandas:** Formal analysis, Resources, Funding acquisition. **Farhan Mohamed:** Methodology, Software. **Faezah Mohd Salleh:** Conceptualization, Methodology, Resources, Writing – review & editing, Supervision, Funding acquisition.

## Declaration of competing Interest

The authors declare that they have no known competing financial interests or personal relationships that could have appeared to influence the work reported in this paper.

## Appendix Supplementary materials

Image, application 1

Image, application 2
